# Systemic Treatment of Metastatic Sertoli Cell Tumor

**DOI:** 10.7759/cureus.104386

**Published:** 2026-02-27

**Authors:** Pedro Serrano, Pedro Barros, Marco Dores, Aníbal Coutinho

**Affiliations:** 1 Department of Urology, Hospital de Faro, Faro, PRT

**Keywords:** hormonal therapy, metastatic disease, multidisciplinary approach, rare testicular neoplasm, sertoli cell tumor

## Abstract

We report the case of a 52-year-old man with a metastatic right testicular Sertoli cell tumor. The patient presented with fatigue, anorexia, unintentional weight loss, and bilateral gynecomastia. Staging computed tomography revealed pleural, hepatic, and bone metastases. Laboratory evaluation demonstrated markedly elevated estradiol and testosterone levels with suppressed gonadotropins. Histopathological analysis of a metastatic soft-tissue lesion confirmed the diagnosis. A multidisciplinary approach combining BEP (bleomycin, etoposide, and cisplatin) chemotherapy with hormonal therapy using letrozole and goserelin was initiated. Despite treatment, disease progression was observed, and enrollment in a clinical trial was considered. The patient ultimately died one month later due to respiratory failure. This case highlights the aggressive behavior of metastatic Sertoli cell tumors and underscores the challenges in managing rare, hormone-producing, treatment-refractory testicular neoplasms.

## Introduction

Sertoli cell tumors are rare testicular neoplasms belonging to the sex cord-stromal tumor group, accounting for less than 1% of all testicular tumors [1]. In contrast to germ cell tumors, which represent the vast majority of testicular malignancies and are typically highly chemosensitive, sex cord-stromal tumors exhibit distinct biological behavior, variable endocrine activity, and generally limited responsiveness to platinum-based chemotherapy [2,3].

Clinically, Sertoli cell tumors may present as a painless testicular mass but can also manifest with endocrine disturbances due to hormone production, including gynecomastia, precocious puberty, or other signs of estrogen or androgen excess. While most cases follow a benign course, a minority behave aggressively, with metastatic potential and poor prognosis once distant spread occurs [2].

Due to their rarity and the absence of standardized treatment protocols for advanced disease, the management of metastatic Sertoli cell tumors remains challenging and is largely based on case reports and small series [4].

## Case presentation

**Figure 1:**
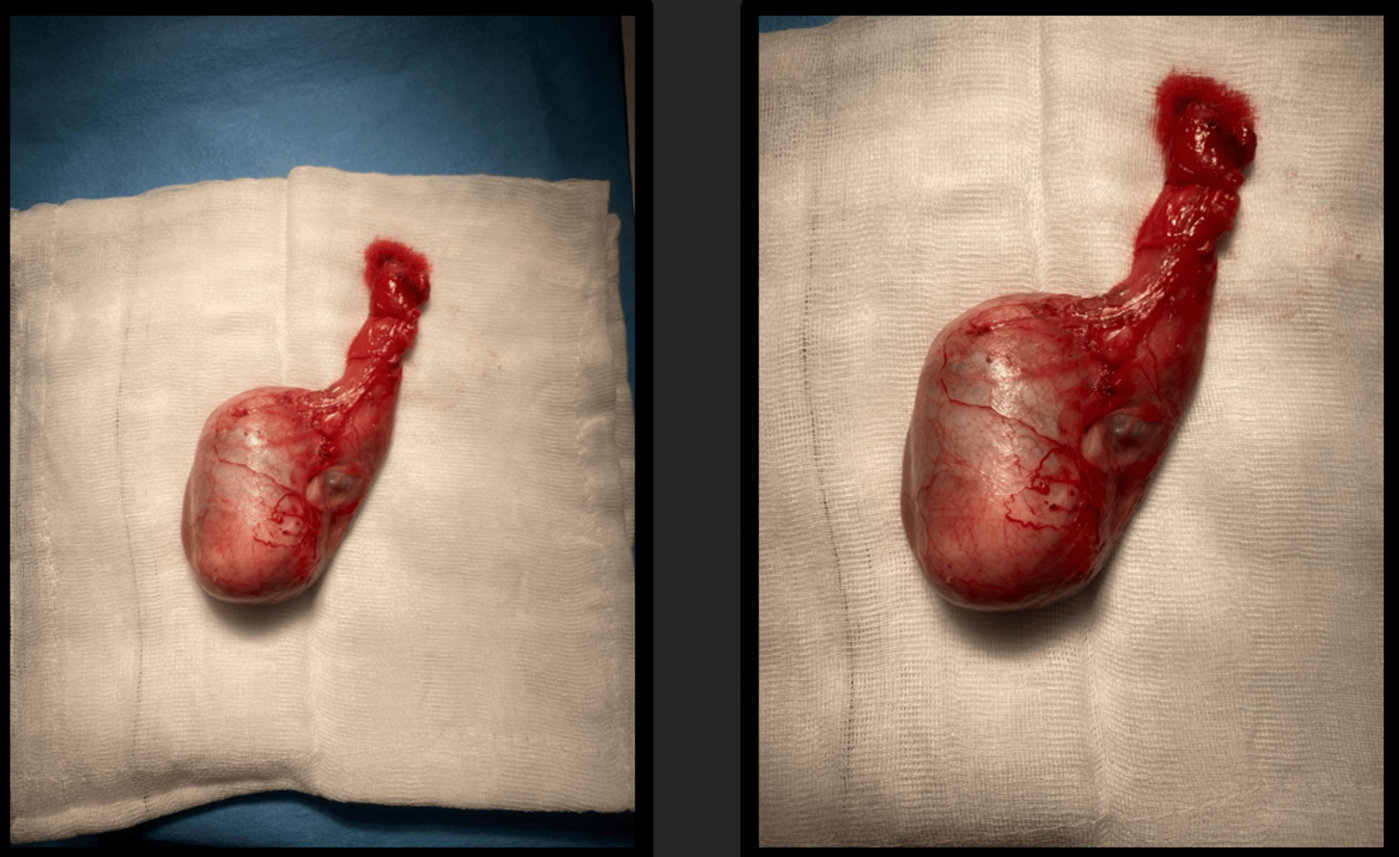
Radical orchiectomy specimen (2024).

We present the case of a 52-year-old married male taxi driver with a history of right radical inguinal orchiectomy in 2024 for a solid intratesticular mass suspicious for malignancy on imaging, in accordance with standard management of testicular tumors. Intraoperatively, a firm right-sided intratesticular lesion was identified without gross local invasion (Figure 1).

Histopathological examination revealed a well-demarcated intratesticular neoplasm measuring 2.9 cm in greatest dimension. On gross examination, the tumor appeared heterogeneous and whitish, with calcified and edematous areas, and it was apparently confined to the testicular parenchyma.

Microscopically, the neoplasm was composed of trabecular and solid nests of cells with abundant eosinophilic cytoplasm embedded in a fibromyxoid stroma containing microcalcifications. The tumor cells exhibited round-to-oval nuclei with marked cytologic atypia and prominent nucleoli. Mitotic activity was identified (4 mitoses per 10 high-power fields).

Immunohistochemical analysis demonstrated positivity for inhibin, calretinin, and S100 protein, with negativity for SALL4, Melan-A, cytokeratin AE1/AE3, β-catenin (membranous staining only), and synaptophysin. These morphological and immunophenotypic findings were consistent with a large cell calcifying Sertoli cell tumor (LCCSCT), classified within sex cord-stromal tumors according to the 2022 WHO Classification of Tumors of the Urinary and Male Genital Systems. The diagnosis was rendered by an experienced genitourinary pathologist. Following orchiectomy, the patient was placed under surveillance.

**Figure 2:**
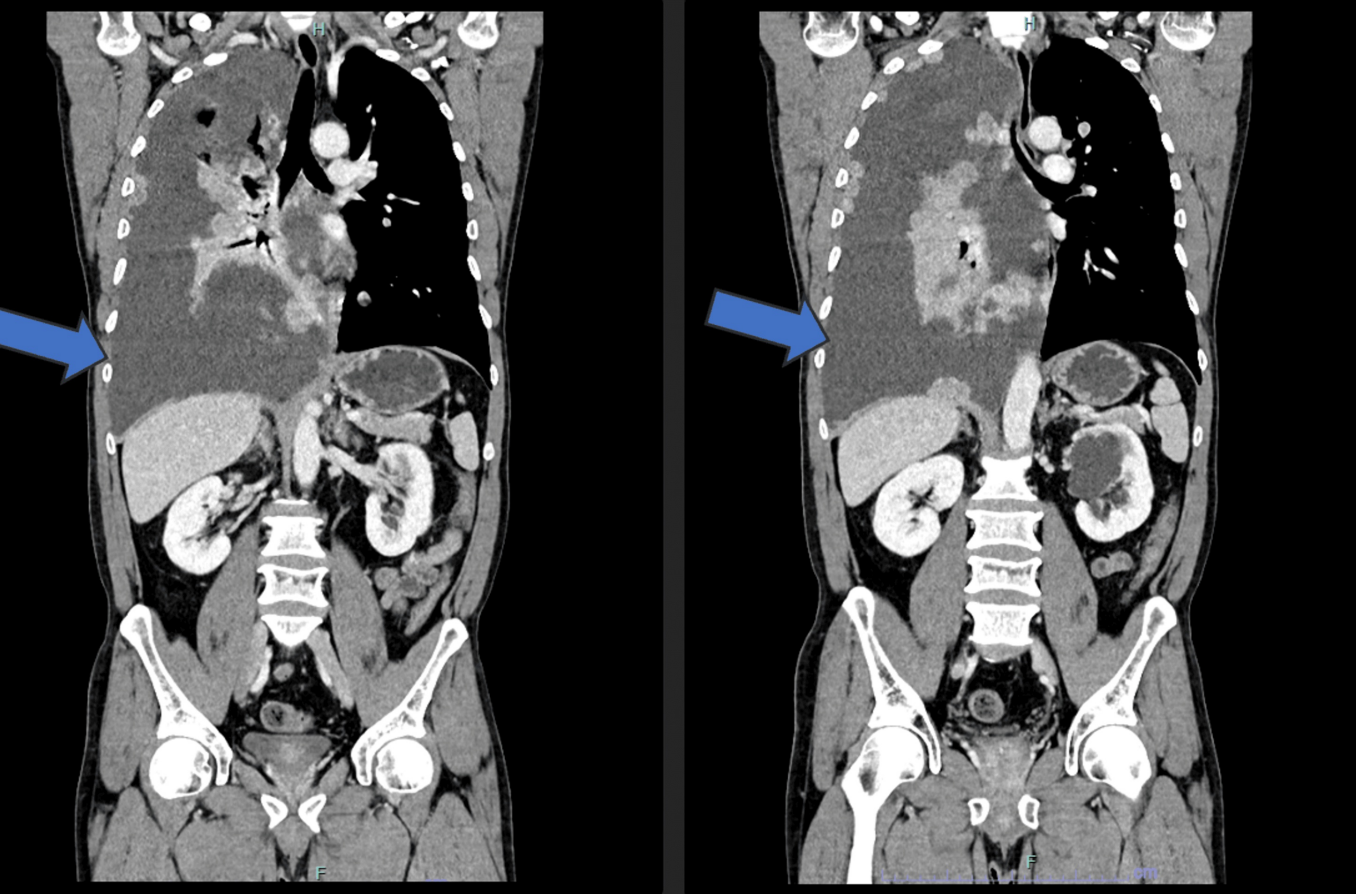
Thoracoabdominopelvic computed tomography (CT) showing metastatic disease.

**Figure 3:**
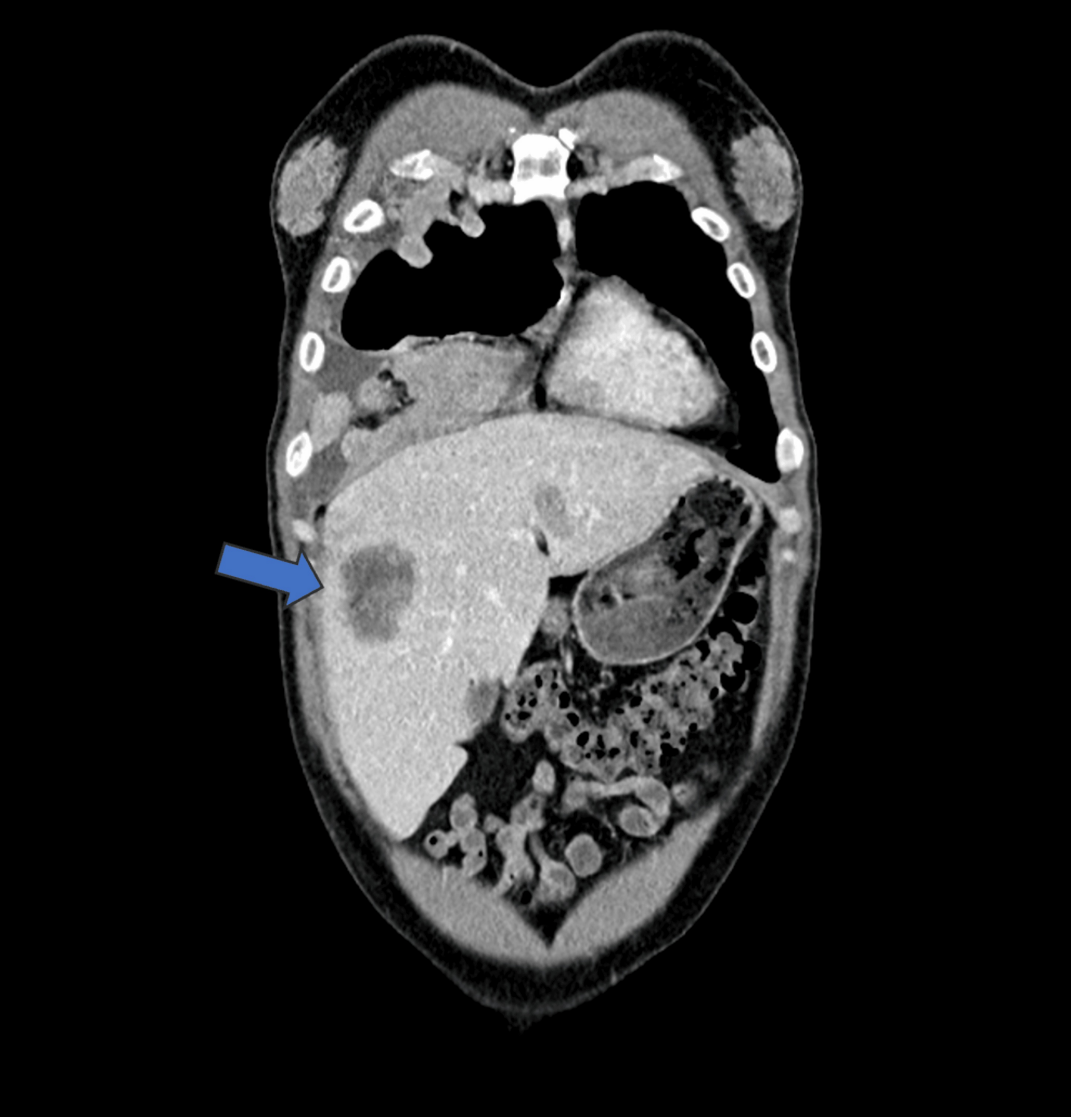
Thoracoabdominopelvic computed tomography (CT) demonstrating a right pleural effusion and hepatic metastases.

In 2025, the patient presented to the emergency department with complaints of fatigue, anorexia, and a significant unintentional weight loss of 4 kg over the preceding two months. A physical examination revealed bilateral gynecomastia [1]. Staging computed tomography (CT) demonstrated metastatic disease, including right pleural effusion, hepatic, and bone metastases, with a destructive lesion involving the left costal arch (Figures 2, 3).

**Figure 4:**
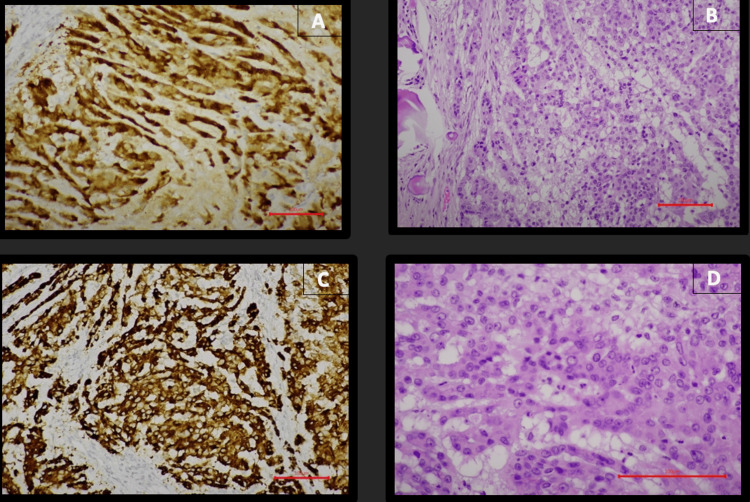
Histopathological findings: (A) Calretinin immunostaining, 200×; (B) Hematoxylin and eosin (H&E), 200×; (C) Inhibin immunostaining, 200×; (D) H&E, 400×, showing marked cellular atypia.

A biopsy of a soft-tissue lesion confirmed metastatic disease originating from the Sertoli cell tumor (Figure 4) [2].

Concomitantly, the patient underwent multiple thoracenteses for symptomatic relief of a large associated pleural effusion [2].

Laboratory evaluation revealed marked hormonal imbalance (Table 1). Serum beta-human chorionic gonadotropin (β-hCG) and alpha-fetoprotein (AFP) levels were within normal limits, helping exclude nonseminomatous germ cell tumors.

**Table 1:** Laboratory evaluation at diagnosis. All values were obtained from serum samples at the time of diagnosis. Reference ranges are for adult males. FSH: Follicle-stimulating hormone; LH: Luteinizing hormone.

Test	Result	Unit	Reference Range
Estradiol	>3000	pg/mL	10–40
Testosterone	>1500	ng/dL	300–1000
Prolactin	92.5	ng/mL	4–​​​​​​​15
FSH	0.30	IU/L	1.5–​​​​​​​12.4
LH	0.30	IU/L	1.2–​​​​​​​8.6

Despite elevated prolactin levels, the patient did not exhibit galactorrhea. Macroprolactin was not specifically excluded using polyethylene glycol (PEG) precipitation testing. Next-generation sequencing (NGS) was performed on tumor tissue to identify potential therapeutic targets and to evaluate for Serine/Threonine Kinase 11 (STK11) mutation in the context of suspected Peutz-Jeghers syndrome, given the association of Sertoli cell tumors with gynecomastia and mucocutaneous pigmentation, although the clinical presentation was not entirely typical. No pathogenic or clinically actionable variants were identified. The results were therefore considered inconclusive in the absence of detectable driver mutations [1].

The case was discussed in a multidisciplinary tumor board. Given the limited responsiveness of metastatic Sertoli cell tumors to conventional chemotherapy, particularly when compared to seminomatous germ cell tumors, and the guarded prognosis described in the literature, systemic treatment was initiated.

Platinum-based chemotherapy with bleomycin, etoposide, and cisplatin (BEP regimen) was administered as first-line systemic therapy for metastatic disease. Following multidisciplinary consensus, and given the markedly elevated estradiol and testosterone levels, hormonal therapy with letrozole (an aromatase inhibitor) and goserelin (a GnRH agonist) was added to the BEP chemotherapy regimen [4]. This combined strategy aimed to reduce peripheral aromatization of androgens while simultaneously suppressing gonadotropin-driven steroidogenesis in an attempt to control disease progression [4]. Letrozole was selected based on limited evidence of response to aromatase inhibitors in Sertoli cell tumors, as described in isolated case reports in the literature [5].

Follow-up CT demonstrated disease progression, with enlargement of hepatic and pleural metastatic lesions [4]. Enrollment in early-phase clinical trials evaluating novel targeted agents was proposed, including studies investigating MTAP-deleted solid tumors, as current literature suggests that novel targeted therapies and immunotherapeutic approaches may represent promising options for rare and chemotherapy-resistant tumors [6].

Due to the patient's refusal to participate in clinical trials outside Portugal, second-line chemotherapy with the TIP (paclitaxel, ifosfamide, and cisplatin) regimen was initiated [4]. The patient ultimately died one month later due to severe respiratory failure [7].

## Discussion

Metastatic Sertoli cell tumors are associated with a poor prognosis, largely due to their limited responsiveness to conventional chemotherapy [2,3]. In this case, despite the combined use of BEP chemotherapy and hormonal therapy with letrozole and goserelin, disease progression was observed, consistent with previously reported outcomes [6].

Although LCCSCTs are frequently described as indolent and are often associated with genetic syndromes such as Peutz-Jeghers syndrome or Carney complex, sporadic cases with malignant behavior have been reported. Histopathological features suggestive of malignancy include significant cytologic atypia, increased mitotic activity, tumor size greater than 4 cm, necrosis, and invasive growth. In the present case, despite a tumor size below 4 cm, marked cytologic atypia and a mitotic rate of 4 mitoses per 10 high-power fields were observed. The subsequent development of distant metastases ultimately confirmed its aggressive biological behavior.

Although no standardized systemic therapy exists for metastatic Sertoli cell tumors, platinum-based regimens such as BEP are often selected by extrapolation from germ cell tumor protocols, given the absence of prospective trials in sex cord-stromal neoplasms. Similarly, second-line regimens such as TIP are derived from salvage strategies used in refractory germ cell tumors, although evidence supporting their efficacy in sex cord-stromal tumors remains low [1,4].

Published metastatic cases are described with survival often measured in months once distant metastases are detected, especially in the presence of visceral involvement. Hormone-producing tumors may initially respond biochemically to endocrine manipulation; however, durable oncologic control remains uncommon. This evidence remains limited and is largely derived from isolated case reports [6].

These observations highlight the aggressive nature of metastatic Sertoli cell tumors. Management remains challenging because these tumors are rare and lack standardized systemic treatment options [5]. Emerging therapeutic strategies, including immunotherapy and targeted treatments, are of increasing interest. Small series suggest that immune checkpoint inhibitors may provide clinical benefit in refractory metastatic sex cord-stromal tumors, although data remain scarce [7]. Molecular profiling may identify actionable alterations, enabling future precision oncology approaches [8].

Given the absence of consensus guidelines and the heterogeneity of these tumors, early multidisciplinary discussion is essential. Referral to specialized centers and consideration of clinical trial enrollment should be strongly encouraged whenever feasible, as these strategies may offer the best potential for clinical benefit in this otherwise treatment-refractory condition [7-10].

## Conclusions

Metastatic Sertoli cell tumors represent a rare and aggressive form of testicular neoplasia with limited responsiveness to conventional chemotherapy. This case illustrates the diagnostic complexity, profound hormonal disturbances, and therapeutic challenges associated with advanced disease.

Despite a multidisciplinary approach combining chemotherapy and hormonal therapy, rapid disease progression occurred, underscoring the poor prognosis of metastatic Sertoli cell tumors. Early referral to specialized centers and consideration of clinical trial enrollment are essential, as emerging targeted and immune-based therapies may offer future therapeutic opportunities for this rare, treatment-refractory malignancy.
